# Mortality and its associated factors among mechanically ventilated adult patients in the intensive care units of referral hospitals in Northwest Amhara, Ethiopia, 2023

**DOI:** 10.3389/fmed.2024.1345468

**Published:** 2024-07-01

**Authors:** Eyob Eshete Tadesse, Ambaye Dejen Tilahun, Nurhusein Nuru Yesuf, Teshome Demis Nimani, Tesfaye Ayenew Mekuria

**Affiliations:** ^1^Department of Nursing, College of Health Sciences, Mettu University, Metu, Ethiopia; ^2^Department of Emergency and Critical Care Nursing, School of Nursing, College of Medicine and Health Sciences, University of Gondar, Gondar, Ethiopia; ^3^Department of Surgical Nursing, School of Nursing, College of Medicine and Health Sciences, University of Gondar, Gondar, Ethiopia; ^4^Department of Epidemiology and Biostatistics, School of Public Health, College of Medicine and Health Sciences, Haramaya University, Harar, Ethiopia; ^5^Department of Intensive Care Unit, Madda Walabu University Goba Referral Hospital, Goba, Ethiopia

**Keywords:** Ethiopia, mechanical ventilation, mortality, Northwest Amhara, intensive care unit

## Abstract

**Background:**

Worldwide, nearly half of the patients admitted to intensive care units require ventilatory support. Despite advances in intensive care unit patient management and mechanical ventilator utilization, the odds of mortality among mechanically ventilated patients are higher in resource-limited settings. Little is known about the mortality of patients on mechanical ventilation outside the capital of Ethiopia. This study aimed to assess mortality and its associated factors among mechanically ventilated adult patients in intensive care units.

**Method:**

An institutional-based cross-sectional study was conducted on mechanically ventilated patients in intensive care units from 1 February 2020 to 1 March 2023. A simple random sampling technique was used to select 434 patients’ charts. A data extraction tool designed on the Kobo toolbox, a smartphone data collection platform, was used to collect the data. The data were exported into Microsoft Excel 2019 and then into Stata 17 for data management and analysis. Descriptive statistics were used to summarize the characteristics of the study participants. A bivariable logistic regression was conducted, and variables with *p* ≤ 0.20 were recruited for multivariable analysis. Statistical significance was declared at *p* < 0.05, and the strength of associations was summarized using an adjusted odds ratio with 95% confidence intervals.

**Result:**

A total of 404 charts of mechanically ventilated patients were included, with a completeness rate of 93.1%. The overall proportion of mortality was 62.87%, with a 95% CI of (58.16–67.58). In the multivariable logistic regression, age 41–70 years (AOR: 4.28, 95% CI: 1.89–9.62), sepsis (AOR: 2.43, 95% CI: 1.08–5.46), reintubation (AOR: 2.76, 95% CI: 1.06–7.21), and sedation use (AOR: 0.41, 95% CI: 0.18–0.98) were found to be significant factors associated with the mortality of mechanically ventilated patients in the intensive care unit.

**Conclusion:**

The magnitude of mortality among mechanically ventilated patients was high. Factors associated with increased odds of death were advanced age, sepsis, and reintubation. However, sedation use was a factor associated with decreased mortality. Healthcare professionals in intensive care units should pay special attention to patients with sepsis, those requiring reintubation, those undergoing sedation, and those who are of advanced age.

## Introduction

Worldwide, the number of patients needing mechanical ventilation (MV) in intensive care units (ICUs) is rising, especially among the elderly and patients with comorbid illnesses (1). Approximately half (40–50%) of patients admitted to the ICU need respiratory assistance with MV (2–6). Among those patients who received MV support in the ICU, a large number of patients, with a rough estimation of approximately 45–60%, will die in the hospital (4, 6, 7). MV is needed in patients with respiratory failure, but it is also associated with increased morbidity and mortality (8–10).

ICU expenses are significantly influenced by MV (11, 12), which accounts for a 25.8% increase in the daily costs of ICU care and accounts for approximately €1,580 for a single ventilated ICU day (12).

In contrast to high-income environments, the mortality rate of patients on MV among developing and low-income countries is higher (13–16). This can be related to the young and underdeveloped nature of intensive care medicine in these areas (17), as well as the lack of trained staff, equipment, and supply material resources (16, 17). According to a review of some studies, the mortality of MV patients ranges from 40.9 to 73.5% in Africa (14, 16, 18, 19). Previous studies conducted in Ethiopia revealed that the magnitude of mortality among mechanically ventilated patients ranges from 28.6 to 60.7% (20–23).

According to studies conducted globally, age (16, 21, 24, 25), sex (26, 27), inotrope and vasopressor use (7, 10, 20, 28, 29), increased duration on MV (10, 20, 23, 30), low serum albumin level (22, 25), decreased Glasgow coma scale score during ICU admission (22), comorbidity (14, 23, 25, 26, 31), need for dialysis (23, 25), multiple organ dysfunction syndrome (MODS) (30), sequential organ failure assessment (SOFA) score (7), acute physiology and chronic health evaluation (APACHE II) score (25, 32), positive end expiratory pressure (PEEP) (33), organ failure (32, 34), admission diagnosis (22, 34), sepsis (32, 35), readmission (36), reintubation (14), tracheostomy use (10, 29, 37), and sedation use (14, 20) were significant factors associated with mortality of mechanically ventilated patients in the ICU. Nevertheless, some of the severity scores, such as APACHE II and SOFA scores, are not applicable in the ICUs of our study settings yet.

Despite advances in the management of patients in the ICU and growing improvements in MV supply and utilization, the odds of mortality among critically ill patients receiving mechanical ventilation support remained higher than their non-ventilated counterparts (38). However, mortality was estimated to be higher in low-resource areas; most of the studies conducted in Ethiopia were concentrated in the capital city, where infrastructure is relatively better. The effect of management factors, such as initial ventilatory settings and reintubation, was also not well studied in those studies. Very little is known about the magnitude of mortality among patients on MV in the peripheral hospitals; thus, this study is aimed at assessing the mortality and its associated factors among mechanically ventilated adult patients in the ICUs of Northwest Amhara referral hospitals.

## Materials and methods

### Study design and period

An institution-based cross-sectional study design was employed through a review of the medical records of patients who were admitted from 1 February 2020 to 1 March 2023. The data were extracted from 10 April to 28 May 2023.

### Study setting

The study was carried out at adult intensive care units of referral hospitals in Northwest Amhara, Ethiopia. In the northwest part of the Amhara region, there are five referral hospitals, including the University of Gondar Comprehensive Specialized Hospital (UOGCSH), Felege Hiwot Comprehensive Specialized Hospital (FHCSH), Debre Markos Comprehensive Specialized Hospital (DMCSH), Tibebe Gihon Comprehensive Specialized Hospital (TGCSH), and Debre Tabor Comprehensive Specialized Hospital (DTCSH). The catchment area for each referral hospital is thought to contain 5–7 million people (39). TGCSH is one of the teaching hospitals in Northwest Amhara, located in Bahirdar City, the capital of Amhara regional state, which is 565 km from the capital, Addis Ababa. There are two intensive care units (pediatric and adult). The adult ICU was equipped with 9 beds, 4 functioning mechanical ventilators, 7 patient monitors, and one bedside ultrasound. This unit is staffed with two anesthesiologists, internal medicine specialists and subspecialists, trained nurses, and medical and surgical residents (40). The pediatric ICU has two beds (41).

FHCSH is also the other referral hospital in Bahirdar City. The adult ICU is one of the 13 wards it has, where critically ill patients are admitted (41). Currently, it has 10 beds and 4 MVs. The UOGCSH is found in Gondar town, 700 km from Addis Ababa. UOGCSH started critical care service in 2011 with a four-bed ICU capacity, two motorized ventilators, one defibrillator, four non-invasive hemodynamic monitoring devices, and one ultrasound machine (42), and currently, it has four ICU departments divided based on specialty: medical ICU, surgical ICU, pediatrics ICU, and neonatal ICU. The adult medical and surgical ICUs of UOGCSH have 22 beds, 11 MVs, 22 monitors, 1 portable x-ray machine, 2 ultrasound machines, and 1 dialyzer machine. DTCSH is found in Debre Tabor town, the capital of the South Gondar zone. It is located approximately 665 km from the capital city of Ethiopia, Addis Ababa. It has three ICUs: 1 adult, 1 pediatric, and 1 neonatal. The adult ICU has 6 beds. DMCSH is located in East Gojam, which is located 300 km and 265 km from Addis Ababa and Bahir Dar, the capitals of Ethiopia and the Amhara regional state, respectively (43). The adult ICU has 4 beds, 3 functional mechanical ventilators, and 3 functional monitors.

### Population

All adult patients admitted to the ICU and received mechanical ventilation support at referral hospitals in Northwest Amhara were the source population. All adult patients admitted to the ICU and received mechanical ventilation support at referral hospitals in Northwest Amhara from 1 February 2020 to 1 March 2023 were the study population. All adult patients admitted to the ICU and who received mechanical ventilation support from 1 February 2020 to 1 March 2023 were included in the study. Those patients who were mechanically ventilated for less than 24 h were excluded from the study. Patient charts with variables not recorded like ICU admission and discharge date, MV initiation time, socio-demographics such as age and sex, GCS during admission, and unknown outcome variable (not recorded and the patient left against medical advice or referred to other hospitals) were declared as incomplete and excluded from the study.

### Sample size determination

For the first objective, a single population proportion formula was used to determine the sample size by taking a proportion, 57.1%, from a study in Saint Paul Hospital Millennium Medical College (SPHMMC) (23); d = margin of error, 5%, and Z α/2 = Z score of the 95% confidence level, 1.96.


$$n=\frac{Z_{\left(\alpha /2\right)}^{2}\;p\;\left(1-p\right)}{d^{2}}$$

$$n=\frac{{\left(1.96\right)}^{2}\;\left(0.571\right)\;\left(0.429\right)=377}{{\left(0.05\right)}^{2}}$$

For the second objective, the sample size was calculated as follows for significantly associated factors: sedation use, inotrope use, and duration of stay on MV (20), using Epi-info version 7.2.2.2, and the maximum sample size was 308. When comparing the sample sizes calculated for both objectives, the sample size obtained from the first objective (377) was found to be the highest. So, to get a maximum sample size, the sample size computed for the first objective was used.

A 15% contingency was considered for possible incomplete medical recording and possible lost charts, taking into account that a dead person’s inactive charts would be difficult to access, and the final sample size was 434.

### Sampling technique and procedures

**Figure 1:**
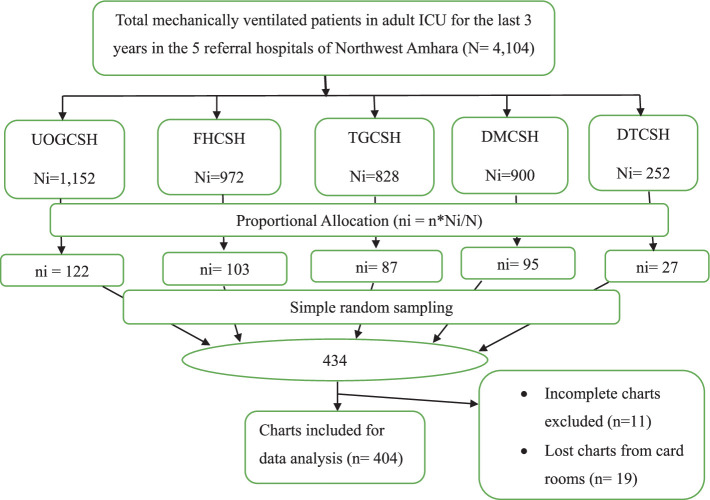
Schematic presentation of sampling procedures used to select mechanically ventilated patients in ICUs of referral hospitals in Northwest Amhara, Ethiopia, 2023.

All the five referral hospitals in the Northwest Amhara were included. After a proportional allocation of sample size made to the respective hospitals based on their 3-year data on MV use, a sampling frame was prepared using computer-generated random numbers by including all chart numbers of patients who were mechanically ventilated from February 2020 to March 2023. Finally, a simple random sampling technique was used to select the samples (Figure 1).

### Operational definitions

**MODS**: Failure of two or more organs at any time in the ICU, depending on ODINS criteria (44).

**Required Hemodialysis**: Presence of two or more findings from the indications of dialysis below:

Electrolyte imbalance: Uncontrolled Hyperkalemia (potassium >6.5 mmoL/L or rising).

Serum sodium level < 115 or > 165 mmoL/L.

Edema (fluid overload): Refractory fluid overload.

Uremia: excessive blood urea nitrogen (BUN) and creatinine levels or any uremic abnormalities such as uremic encephalopathy, uremic pericarditis, and the like.

Acidosis: severe metabolic acidosis with concomitant acute kidney injury, pH < 7.

Intoxication: life-threatening poisoning with a dialyzable drug, such as salicylates, lithium, isopropanol, methanol, or ethylene glycol (45).

**Barotrauma**: Radiographically confirmed pneumothorax, pneumomediastinum, or subcutaneous emphysema that could not be attributed to iatrogenic injury (46).

**Comorbidity** is the co-occurrence of two or more disorders or diseases at the same time (47). Comorbidity was declared if the patient has at least one chronic illness other than the acute indication for MV.

**Incomplete Patient Chart**: Patients’ charts were declared incomplete when they did not consist of complete baseline medical data, specifically for variables not recorded such as ICU admission date and discharge date, MV initiation time, socio-demographics such as age and sex, GCS during admission, and unknown outcome variables (not recorded and patient left against medical advice or referred to other hospitals).

**Hypertension** was defined as a blood pressure reading of systolic ≥130 and diastolic ≥80. At the same time, **Normotensive** was defined as a blood pressure reading of systolic 90–129 and diastolic 60–80 (48).

**Hypotension** was defined as a blood pressure reading of systolic <90 and diastolic <60 (49).

**Tachypnea** was defined as a respiratory rate > 30 breaths per minute (50).

**Bradypnea** was defined as a respiratory rate of <12 breaths per minute (51).

**Sedation use** was defined as having received an intravenous or intramuscular sedative (ketamine, benzodiazepines, dexmedetomidine, barbiturates, or propofol) for any period during the intensive care stay (52). This does not include the sedation used for procedures such as intubation.

**Vasopressor use** was defined as utilizing epinephrine, norepinephrine, vasopressin, dopamine, or phenylephrine (53) during ICU stay.

### Data collection tool and procedures

A data extraction checklist was developed from mechanical ventilation protocols and related literature (10, 16, 20–23). The data extraction tool comprised socio-demographic data, such as age, sex, and residence; clinical characteristics such as admission diagnosis, GCS at admission, serum albumin level, hemoglobin level, sepsis, indication for MV, vital signs, presence and types of comorbidity, presence and types of organ failure, and presence of MODS; and management-related characteristics, such as readmission, reintubation, sedation use, vasopressor use, required dialysis, initial ventilatory settings, and duration of stay in ICU and on MV. Five trained BSc nurses working in emergency wards of the respective hospitals collected the data using the Kobo toolbox, a mobile and tablet-based data collection platform. Four trained MSc nurses working in wards other than the adult ICU supervised the data collection process. The patients’ charts were found by taking the medical record number (MRN) from the log book at the ICU. Then, the charts were extracted from the card rooms of the corresponding hospitals. All randomly selected charts were roughly reviewed, and relevant data were extracted. For patients with readmission and reintubation, we used the last admission and intubation, respectively, to extract the data.

### Data quality control

To control the quality of the data, data collectors and supervisors were trained separately for 2 days about the objectives of the study, confidentiality, and data collection techniques. The relevance of the variables in the tool was verified by consulting experts with a critical care specialty. Before the actual data collection, a preliminary chart review was conducted on 22 (5%) randomly selected charts at UOGCSH to check the accessibility of variables. Accordingly, variables that were repetitively not recorded in the patient recordings (inotrope use and I:E ratio) were excluded from the data extraction checklist. In addition, ventilation for less than 24 h was made as an exclusion criterion due to the absence of important variables for those ventilated for less than 24 h. During data collection, each filled checklist was cross-checked and revised daily by the investigator for completeness. Data cleaning was performed before analysis.

### Data processing and analysis

After completion of data collection, the data were exported from the Kobo Toolbox Server into Microsoft Excel 2019 for data cleaning and management, and then it was exported to Stata 17 for data management and analysis. The descriptive statistics were described using texts, frequency tables, percentages, and graphs, whereas mean with standard deviation and median with interquartile ranges were used for continuous variables after the data distribution was checked by histogram and skewness and kurtosis tests to characterize study participants. Mean imputation was used to manage missing values for the continuous variables, baseline serum albumin level, baseline hemoglobin level, and temperature at the initiation of MV. The category of interest (died) was coded as 1 and survived was coded as 0. All relevant variables were included. The chi-square assumption test was done for categorical independent variables. Multicollinearity was checked by the variance inflation factor (VIF) and the variables; the presence of complication and VAP were excluded due to VIF > 10. Bivariable analysis was conducted using the binary logistic regression model to determine the association between each independent variable and the outcome variable. Accordingly, variables with a *p*-value of ≤0.20 were considered for further analysis (multivariable analysis) to identify the net effect of each variable on the outcome variable. Finally, statistical significance was declared at *p* < 0.05, and the strength of associations was summarized using an adjusted odds ratio (AOR) with 95% confidence intervals (CI). The goodness of model fitness was checked using the Hosmer–Lemeshow goodness test.

## Results

### Socio-demographic characteristics of the study participants

A total of 404 charts of mechanically ventilated patients in the ICUs were included in the study, with a completeness rate of 93.1%. More than half (59.16%) of the patients were aged between 18 and 40 years, with a median age of 35.5 (IQR: 25–53). Two hundred twenty-five (55.69%) of the patients were male, and approximately 59.41% were rural residents (Table 1).

**Table 1:** Socio-demographic characteristics of mechanically ventilated patients in the ICUs of referral hospitals in Northwest Amhara, Ethiopia, 2023 (*n* = 404).

Variables	Categories	Frequency	Percent (%)
Age	18–40	239	59.16
	41–70	132	32.67
	>70	33	8.17
Sex	Male	225	55.69
	Female	179	44.31
Residence	Rural	240	59.41
	Urban	164	40.59

### Clinical characteristics of the study participants

The main admission diagnosis for one-third of the study participants was respiratory, categorically. One hundred seventy-four (43.07%) of the study participants had a GCS of less than or equal to 8 or 7 with intubation (7T) during admission to the ICU. At the initiation of MV, more than half (62.13, 54.46, and 53.96%) of the patients were tachycardic, tachypneic, and hypoxic, respectively. Respiratory failure was the most common indication for MV (45.3%). Among the total study participants, the majority (81.44%) of them had at least one organ failure, and more than half (51.98%) had MODS. Only 87 (21.53%) patients had developed a complication, with VAP occupying the highest proportion (67.8%) (Table 2).

**Table 2:** Clinical characteristics of mechanically ventilated patients in the ICUs of referral hospitals in Northwest Amhara, Ethiopia, 2023 (*n* = 404).

Variables	Categories	Frequency (*N* = 404)	Percent (%)
Main admission Diagnosis	Respiratory	136	33.66
	Cardiovascular	40	9.9
	Neurologic	80	19.8
	Renal	21	5.2
	Obstetric and gynecologic	26	6.44
	Surgical (non-trauma)	31	7.67
	Trauma	70	17.33
Presence of comorbidity	No	220	54.46
	Yes	184	45.54
Type of comorbidity (*N* = 184)	Chronic kidney disease	31	16.8
	Asthma	22	11.9
	Diabetes mellitus	33	17.9
	Hypertension	50	27.2
	COPD	9	4.9
	CHF	35	19
	HIV/AIDS	33	17.9
	Stroke	21	11.4
	Others*	9	4.9
GCS at ICU admission	≤8/7 T	174	43.07
	9–12/≥8 T	92	22.77
	13–15	138	34.16
Baseline serum albumin level	>2 g/dL	313	77.48
	≤2 g/dL	91	22.52
Baseline Hemoglobin level	≤7	38	9.41
	7.1–10	70	17.33
	10.1–13.5	198	49
	≥13.5	98	24.26
Blood pressure at initiation of MV	Hypotensive	129	31.93
	Hypertensive	84	20.8
	Normal	191	47.27
Heart rate at initiation of MV	Tachycardia	251	62.13
	Bradycardia	6	1.48
	Normal	147	36.39
Respiratory rate at initiation of MV	Tachypnea	220	54.46
	Bradypnea	5	1.24
	Normal	179	44.3
Temperature at initiation of MV	Normothermia	226	55.94
	Hypothermia	43	10.64
	Hyperthermia	135	33.42
Oxygen saturation at initiation of MV	Non-hypoxic	186	46.04
	Hypoxic	218	53.96
Indication for MV	Neurologic/coma/airway protection	145	35.9
	Neuromuscular diseases	40	9.9
	Respiratory failure	183	45.3
	Cardiovascular failure/shock	36	8.9
Type of respiratory failure (*N* = 183)	Type 1	152	83.06
	Type 2	31	16.94
Organ failure presence	No	75	18.56
	Yes	329	81.44
Type of organ failure (*N* = 329)	Renal failure	90	27.35
	Respiratory failure	202	61.4
	Neurologic failure	140	42.5
	Cardiovascular failure	133	40.4
	Hematologic failure	34	10.3
	Hepatic failure	6	1.8
	Infectious failure	12	3.65
MODS	No	194	48.02
	Yes	210	51.98
Sepsis	No	242	59.9
	Yes	162	40.1
Complication developed	No	317	78.47
	Yes	87	21.53
Type of complication (*N* = 87)	VAP	59	67.8
	Barotrauma	8	9.2
	Pulmonary embolism	3	3.4
	ARDS	13	14.9
	Post-extubation stridor	5	5.7

Others*- Chronic liver disease, hepatitis, valvular heart disease, and epilepsy.

### Management-related characteristics of the study participants

The median duration of ventilation for the study participants was 7 days (IQR: 3–12). Most of the patients (71.3%) had used sedatives, and nearly one-fourth (21.29%) of the patients were reintubated. The majority (87.87%) of the access to the airway was endotracheal tube and 46.53% of the patients were initiated by pressure control with assisted control mode. The median lengths of stay in the ICU and hospital for the study participants were 9 days (IQR: 5–15) and 12 days (IQR: 7–19), respectively. The mean initial Fi02 of the study participants was 83.28% ± 21.47 (Table 3).

**Table 3:** Management-related characteristics of mechanically ventilated patients in the ICUs of referral hospitals in Northwest Amhara, Ethiopia, 2023 (*n* = 404).

Variables	Categories	Frequency (*N* = 404)	Percent (%)
Readmission	No	342	84.65
	Yes	62	15.35
Intubation time	Working day, daytime	174	43.07
	Working day, nighttime	137	33.9
	Weekend, daytime	63	15.6
	Weekend, nighttime	30	7.43
Reintubation	No	318	78.71
	Yes	86	21.29
Airway access	ET tube	355	87.87
	Tracheostomy	49	12.13
Initial mode of ventilation	PC/ AC	188	46.53
	VC/ AC	157	38.86
	SIMV	44	10.9
	CPAP	15	3.71
Duration of ventilation	1–10	272	67.33
	11–20	82	20.3
	>20	50	12.37
Length of stay in ICU	1–10	223	55.2
	11–20	112	27.72
	>20	69	17.08
Required hemodialysis	No	312	77.23
	Yes	92	22.77
Sedative use	No	116	28.7
	Yes	288	71.3
Vasopressor use	No	230	56.9
	Yes	174	43.1
Extubation time	Working day, daytime	192	47.5
	Working day, nighttime	100	24.8
	Weekend, daytime	47	11.6
	Weekend, nighttime	65	16.1

### Magnitude of mortality among mechanically ventilated adult patients in the ICU

**Figure 2:**
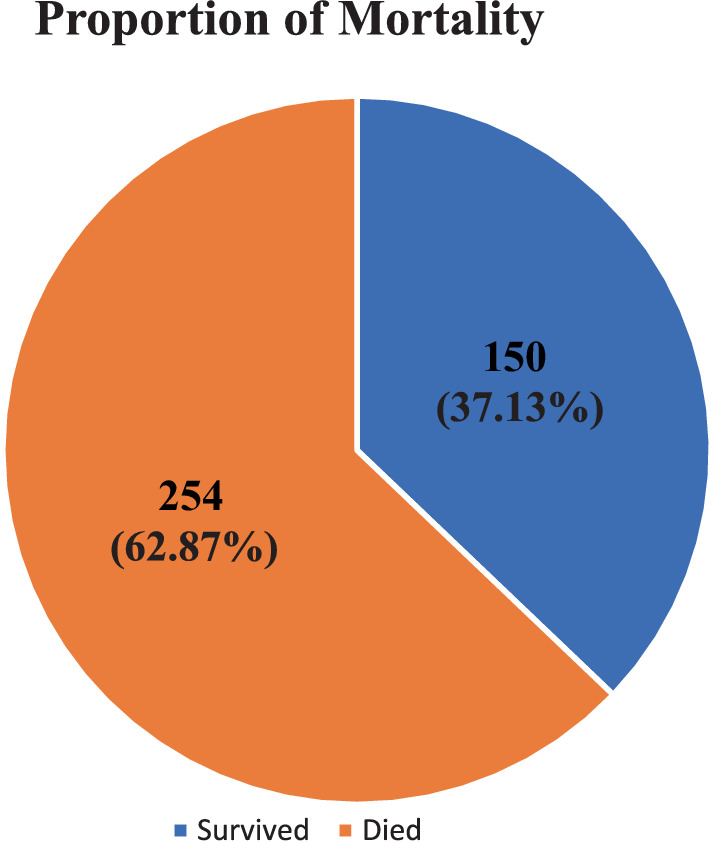
Magnitude of mortality among mechanically ventilated adult patients in the ICUs of referral hospitals in Northwest Amhara, Ethiopia, 2023.

**Figure 3:**
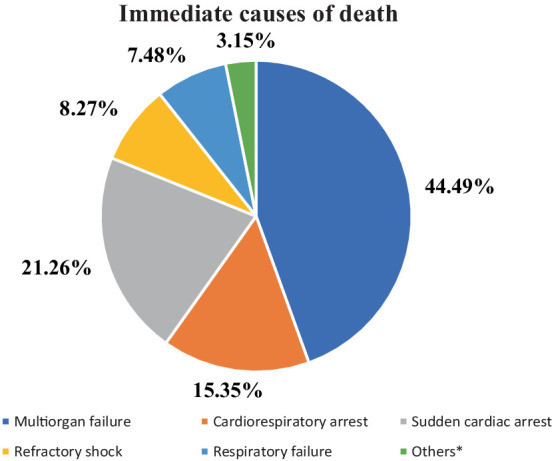
Registered causes of death among mechanically ventilated adult patients at ICUs of referral hospitals in Northwest Amhara, Ethiopia, 2023 (*n* = 254). Others*- Cardiovascular arrest, intracranial hemorrhage, and brain death.

In this study, the proportion of deaths among mechanically ventilated patients in the ICU was 254 (62.87%) with a 95% CI of (58.16–67.58) (Figure 2). The most common immediate cause of death registered was multiorgan failure, which accounts for 44.49% (Figure 3).

### Factors associated with mortality of mechanically ventilated patients

In the multivariable logistic regression analysis, age (41–70), sepsis, reintubation, and sedation use were found to be significant factors associated with the mortality of mechanically ventilated patients at ICUs at a *p*-value <0.05.

Keeping all other variables constant, the odds of mortality among the 41–70 years age group was 4.3 (AOR, 4.28, 95% CI: 1.89–9.62) times higher than those in the 18–40 years age group. While controlling for other variables, the odds of mortality among patients with sepsis was 2.4 (AOR, 2.43, 95% CI: 1.08–5.46) times greater than those without sepsis. Patients who were reintubated were 2.8 (AOR, 2.76, 95% CI: 1.06–7.21) times more likely to die than those non-reintubated patients, holding all other factors constant. However, using sedation decreased the odds of mortality by 59% (AOR, 0.41; 95% CI: 0.18–0.98) compared to patients not taking sedation, while other covariates remained the same (Table 4). The Hosmer–Lemeshow goodness of model fitness showed that the model is good-fitted at *p* = 0.4510 (Table 5). The mean VIF was found to be 2.28 (Table 6).

**Table 4:** Results of bivariable and multivariable logistic regression analysis of factors associated with the mortality of mechanically ventilated patients in adult ICUs of referral hospitals in Northwest Amhara, Ethiopia, 2023 (*n* = 404).

Variables	Mortality status		COR (95% CI)	AOR (95% CI)
	Died *n* (%)	Survived *n* (%)		
Age in years				
18–40	126 (52.72)	113 (47.28)	1	1
41–70	99 (75.0)	33 (25.0)	2.69 (1.68–4.29)	4.28 (1.89–9.62) *
>70	29 (87.88)	4 (12.12)	6.50 (2.22–19.07)	3.56 (0.73–17.39)
Serum albumin level				
>2 g/dL	179 (57.19)	134 (42.81)	1	1
≤ 2 g/dL	75 (82.42)	16 (17.58)	3.51 (1.96–6.29)	0.96 (0.38–2.43)
Blood pressure at the initiation of MV				
Hypotensive	103 (79.84)	26 (20.16)	4.54 (2.71–7.60)	2.13 (0.80–5.69)
Hypertensive	62 (73.81)	22 (26.19)	3.23 (1.84–5.67)	1.55 (0.63–3.81)
Normotensive	89 (46.6)	102 (53.4)	1	1
Admission GCS				
≤8/7 T	132 (75.86)	42 (24.14)	3.97 (2.45–6.43)	1.95 (0.68–5.59)
9–12/≥8 T	61 (66.3)	31 (33.7)	2.48 (1.44–4.29)	2.32 (0.96–5.62)
13–15	61 (44.2)	77 (55.8)	1	1
Admission diagnosis				
Respiratory	81 (59.56)	55 (40.44)	1	1
Cardiovascular	29 (72.5)	11 (27.5)	1.79 (0.82–3.88)	0.80 (0.19–3.28)
Neurologic	52 (65)	28 (35)	1.26 (0.71–2.24)	0.55 (0.15–1.99)
Renal	18 (85.71)	3 (14.29)	4.07 (1.14–14.50)	2.42 (0.39–15.17)
Obstetric/gynecologic	18 (69.23)	8 (30.77)	1.53 (0.62–3.76)	0.32 (0.06–1.62)
Surgical (non-trauma)	15 (48.39)	16 (51.61)	0.64 (0.29–1.39)	0.66 (0.13–3.27)
Trauma	41 (58.57)	29 (41.43)	0.96 (0.53–1.72)	0.65 (0.18–2.39)
Comorbidity				
No	103 (46.82)	117 (53.18)	1	1
Yes	151 (82.07)	33 (17.93)	5.19 (3.28–8.24)	2.47 (0.99–6.15)
Diabetes mellitus				
No	229 (61.73)	142 (38.27)	1	
Yes	25 (75.76)	8 (24.24)	1.94 (0.85–4.41)	0.72 (0.19–2.75)
Hypertension				
No	211 (59.6)	143 (40.4)	1	1
Yes	43 (86)	7 (14)	4.16 (1.82–9.51)	1.91 (0.47–7.81)
Congestive heart failure				
No	226 (61.25)	143 (38.75)	1	1
Yes	28 (80)	7 (20)	2.53 (1.08–5.95)	0.85 (0.19–3.75)
Hemoglobin level				
≤7	28 (73.68)	10 (26.32)	2.28 (1.00–5.20)	1.34 (0.27–6.78)
7.1–10	45 (64.29)	25 (35.71)	1.47 (0.78–2.76)	0.92 (0.29–2.89)
10.1–13.5	127 (64.14)	71 (35.86)	1.46 (0.89–2.39)	0.81 (0.36–1.82)
>13.5	54 (55.1)	44 (44.9)	1	1
Indication for MV				
Neurologic/coma/Airway protection	87 (60)	58 (40)	0.93 (0.59–1.45)	0.38 (0.08–1.67)
Neuromuscular diseases	25 (62.5)	15 (37.5)	1.03 (0.51–2.09)	5.51 (0.89–33.98)
Respiratory failure	113 (61.75)	70 (38.25)	1	1
Cardiovascular failure/shock	29 (80.56)	7 (19.44)	2.57 (1.07–6.17)	0.62 (0.10–3.83)
Last temperature before initiation of MV				
Normothermia	131 (57.96)	95 (42.04)	1	1
Hypothermia	35 (81.4)	8 (18.6)	3.17 (1.41–7.15)	2.37 (0.72–7.77)
Hyperthermia	88 (65.19)	47 (34.81)	1.36 (0.87–2.11)	0.79 (0.38–1.66)
Organ failure				
No	26 (34.67)	49 (65.33)	1	1
Yes	228 (69.3)	101 (30.7)	4.25 (2.50–7.22)	2.12 (0.43–10.54)
Renal failure				
No	176 (56.05)	138 (43.95)	1	1
Yes	78 (86.67)	12 (13.33)	5.10 (2.67–9.74)	1.03 (0.20–5.16)
Respiratory failure				
No	120 (59.41)	82 (40.59)	1	1
Yes	134 (66.34)	68 (33.66)	1.35 (0.90–2.02)	0.70 (0.17–2.95)
Neurologic failure				
No	140 (53.03)	124 (46.97)	1	1
Yes	114 (81.43)	26 (18.57)	3.88 (2.38–6.34)	3.06 (0.67–14.04)
Cardiovascular failure				
No	141 (52.03)	130 (47.97)	1	1
Yes	113 (84.96)	20 (15.04)	5.21 (3.06–8.87)	1.73 (0.34–8.83)
Hematologic failure				
No	226 (61.08)	144 (38.92)	1	1
Yes	28 (82.35)	6 (17.65)	2.97 (1.20–7.36)	0.99 (0.18–5.65)
MODS				
No	71 (36.6)	123 (63.4)	1	1
Yes	183 (87.14)	27 (12.86)	11.74 (7.13–19.33)	2.96 (0.58–15.15)
Sepsis				
No	118 (48.76)	124 (51.24)	1	1
Yes	136 (83.95)	26 (16.05)	5.49 (3.37–8.97)	2.43 (1.08–5.46) *
Readmission				
No	200 (58.48)	142 (41.52)	1	1
Yes	54 (87.1)	8 (12.9)	4.79 (2.21–10.38)	2.84 (0.88–9.12)
Intubation time				
Working day, daytime	94 (54.02)	80 (45.98)	1	1
Working day, nighttime	92 (67.15)	45 (32.85)	1.74 (1.09–2.77)	1.41 (0.64–3.10)
Weekend, daytime	43 (68.25)	20 (31.75)	1.83 (0.99 3.36)	2.16 (0.77–6.08)
Weekend, nighttime	25 (83.33)	5 (16.67)	4.26 (1.56–11.63)	3.41 (0.79–14.55)
Reintubation				
No	186 (58.49)	132 (41.51)	1	1
Yes	68 (79.07)	18 (20.93)	2.68 (1.52–4.72)	2.76 (1.06–7.21)*
Initial ventilator mode				
PC/AC	107 (56.91)	81 (43.09)	1	1
VC/AC	112 (71.34)	45 (28.66)	1.88 (1.20–2.96)	1.13 (0.53–2.40)
SIMV	23 (52.27)	21 (47.73)	0.83 (0.43–1.60)	1.12 (0.35–3.55)
CPAP	12 (80)	3 (20)	3.03 (0.83–11.08)	2.54 (0.27–24.26)
Initial FIO2	100% (IQR: 60–100)		0.99 (0.98–1.00)	0.99 (0.98–1.01)
Initial PEEP	6 (IQR: 5–8)		0.90 (0.81–0.99)	1.05 (0.88–1.24)
Required hemodialysis				
No	177 (56.73)	135 (43.27)	1	1
Yes	77 (83.70)	15 (16.30)	3.92 (2.16–7.11)	2.90 (0.72–11.65)
Sedation use				
No	92 (79.31)	24 (20.69)	1	1
Yes	162 (56.25)	126 (43.75)	0.34 (0.20–0.56)	0.41 (0.18–0.98) *
Vasopressor use				
No	113 (49.13)	117 (50.87)	1	1
Yes	141 (81.03)	33 (18.97)	4.42 (2.80–6.99)	1.64 (0.61–4.38)

*Statistically significant at *p* < 0.05; 1, reference; FI02, fraction of inspired oxygen; PEEP, positive end expiratory pressure; SIMV, synchronized intermittent mandatory ventilation; CPAP, continuous positive airway pressure; VC/AC, volume controlled assisted control; PC/AC, pressure controlled assisted control; MODS, multiple organ dysfunction syndrome; GCS, Glasgow Coma Scale; IQR, interquartile range; COR, crude odds ratio; AOR, adjusted odds ratio.

**Table 5:** Hosmer–Lemeshow goodness-of-fit test for the multivariable logistic regression analysis of factors associated with the mortality of mechanically ventilated patients in adult ICUs of referral hospitals in Northwest Amhara, Ethiopia, 2023 (*n* = 404).

Number of observations	404
Number of groups	10
Hosmer–Lemeshow chi2 (8)	7.82
Prob > chi2	0.4510

**Table 6:** Results of multicollinearity test for factors associated with the mortality of mechanically ventilated patients in adult ICUs of referral hospitals in Northwest Amhara, Ethiopia, 2023 (*n* = 404).

Variable	Vif	1/Vif
Presence of comorbidity	5.39	0.185691
Respiratory failure	5.20	0.192342
MODS	4.84	0.206509
Cardiovascular failure	4.47	0.223793
Renal failure	4.34	0.230652
Duration of ventilation	3.75	0.266398
Neurologic failure	3.74	0.267292
Length of stay in ICU	3.33	0.300013
Oxygen saturation at initiation of MV	3.15	0.317129
Required hemodialysis	3.15	0.317955
Presence of organ failure	3.05	0.328366
Vasopressor use	2.67	0.374532
GCS at ICU admission	2.30	0.434746
HIV/AIDS	2.25	0.445293
Indication for MV	2.19	0.456595
Chronic kidney disease	2.18	0.457992
Hypertension	2.08	0.480453
Diabetes mellitus	2.03	0.492034
Main admission diagnosis	2.01	0.497492
Asthma	1.99	0.503646
Respiratory rate at initiation of MV	1.98	0.505784
Hematologic failure	1.85	0.539547
Baseline hemoglobin level	1.79	0.558254
Age of the patient	1.79	0.558537
Blood pressure at initiation of MV	1.76	0.568677
Congestive heart failure	1.74	0.573772
Presence of sepsis	1.71	0.584657
Reintubation	1.62	0.618082
Infectious failure	1.61	0.620387
Stroke	1.59	0.628897
Sedative use	1.57	0.635789
Airway access	1.55	0.643840
COPD	1.46	0.684478
Baseline serum albumin level	1.44	0.694144
Extubation time	1.38	0.725461
Initial PEEP	1.37	0.729806
Heart rate at initiation of MV	1.35	0.740303
Initial mode of ventilation	1.29	0.777353
Readmission	1.26	0.795441
Sex of the patient	1.25	0.801868
Temperature at the initiation of MV	1.24	0.805046
Hepatic failure	1.23	0.813025
Residence	1.23	0.815161
Intubation time	1.22	0.817741
Mean Vif	2.28	

## Discussion

In Ethiopia, patients who need mechanical ventilation were nearly five times more likely to die in the ICU than those who do not (38). Therefore, this study aimed to assess mortality and its associated factors among mechanically ventilated adult patients in intensive care units of referral hospitals in Northwest Amhara.

According to this study, the overall proportion of mortality among mechanically ventilated adult patients in the ICU was 62.87%, with a 95% CI of (58.16–67.58). This finding was higher than the observational study conducted in Argentina (44.6%) (35). This discrepancy might have resulted from a lack of standard illness severity scores and mortality predictions such as APACHE, which aid in anticipating mortality and considering special attention. The finding of this study was also higher than the study in Canada (18%) (54). In addition to differences in the quality of ICU care provided, this variation could be related to the difference in the study population; the study from Canada was conducted only on patients with acute respiratory failure, whereas the current study included all diagnoses. In this study, the proportion of mortality was higher than in the study conducted in Japan, 38.8% (55). The discrepancy could be due to exclusion criteria, as the study in Japan excluded patients who were mechanically ventilated for less than 3 days, cancer patients, and patients who stayed more than 60 days in the ICU. This might decrease the proportion since patients with an expected poor prognosis were excluded from the very beginning.

The proportion of mortality found in this study was also higher compared to similar studies in low-income countries. The finding of this study revealed that the mortality proportion of mechanically ventilated patients was greater than the study conducted in Egypt (40.9%) (14). The possible explanation for this discrepancy could be organizational structure since the study in Egypt was conducted specifically in the respiratory ICU, where management of mechanically ventilated patients will be more focused and organized. Similarly, the proportion of deaths found in this current study was higher than in previous studies conducted in Ethiopia; a multicenter study in Addis Ababa (41.7%) (20), SPHMMC (57.1%) (23), and Ayder Hospital, Mekelle (28.6%) (21). The study period, small sample size, lack of trained professionals (56), COVID-19 ICU burden, and the Northern Ethiopian conflict might have contributed to this high mortality. The study in Ayder Hospital, Mekelle, was conducted on relatively small sample sizes (105 samples). In addition to this, the study period in Ayder was free from both COVID-19 and the conflicting burden when ICU admission and the need for MV reached their peak.

However, the findings of this study showed a mortality proportion lower than that of a study conducted in India (83%) (57). This discrepancy could also be due to a difference in the study period; the previous study was conducted from 2013 to 2015, while our study is recent. The other possible justification for this discrepancy might be due to the presentation of the patients in the advanced stage of the disease having received poor or delayed pre-hospital care, leading to a poor outcome in these severely ill patients in the study in India (57).

Despite these discrepancies, this finding was comparable with a second study from a different center in India (67.21%) (34). The possible reason might be the similarity in inclusion criteria. Similar to the current study, the previous study also included patients greater than 18 years of age with all admission diagnoses. Similarly, the mortality proportion in this study was in line with a study conducted in TASH (60.7%) (22). This similarity could be due to similarities in admission diagnosis and indications; in both studies, respiratory problems and respiratory failure were the most common admission diagnoses and indications of MV, respectively. The mortality proportion in this study was in line with a study in Kenya (60.7%) (16). Since both studies are conducted in peripheral hospitals with limited resources, the possible justification for this similarity could be due to the similar demography and socio-economic status of the study settings (56). It was also in line with a study in Cairo, Egypt (64%) (18).

This study revealed that age, presence of sepsis, sedation use, and reintubation were factors significantly associated with the mortality of mechanically ventilated patients in the ICU. Those in the 41–70 years age group had 4.3 times higher odds of mortality than those in the 18–40 years age group. This finding was consistent with previous studies conducted in India (34), Brazil (24), Kenya (16), Taiwan (25), Argentina (35), Addis Ababa, Ethiopia (31), and Mekelle, Ethiopia (21). This could be because older patients are more likely to experience acute respiratory failure, especially those over the age of 65 years (58), or due to declining physiologic reserve and function across multiple organ systems, which increases vulnerability to unfavorable health outcomes (59). Apart from this, it can also be related to a higher comorbidity burden among advanced-age patients (60).

According to our study, sepsis was also found to be a significant factor associated with the mortality of mechanically ventilated patients in ICUs. Patients who had sepsis had 2.43 times greater odds of mortality than those without sepsis. This finding was corroborated by the studies previously conducted in Argentina (35) and southern Brazil (32). The first reason could be that sepsis is a common cause of lung injury and increases lung susceptibility to ventilator-induced lung injury. Despite this fact, the specifics of the management of sepsis-induced lung injury are largely unknown (61). Second, ARDS is a devastating complication of severe sepsis, which is responsible for high mortality (62). Third, in addition to signs of infection, a host’s response to an infection manifests with acute organ dysfunction, and this dysfunction can lead to multiple organ failure, acidosis, and death. Furthermore, sepsis can progress to its subset, septic shock, in which underlying circulatory, cellular, and metabolic abnormalities are profound enough to substantially increase the risk of mortality (63). Last but not least, the lack of sepsis assessment scales, such as quick sepsis-related organ failure assessment (qSOFA) and SOFA in our study settings, could also be a reason for this finding.

Reintubation was also another factor found to be significantly associated with the mortality of mechanically ventilated patients in ICUs. Patients who were reintubated had 2.8 times higher mortality odds than their non-reintubated counterparts. This result was in agreement with studies conducted in Egypt (14), Korea (64), the USA (65), and Brazil (66). This could be justified scientifically, as evidence from the SRMA (67) study revealed that reintubation increases the risk of acquiring VAP, which in turn increases the risk of mortality. Since intubation is an invasive procedure, we cannot deny that the repetitive action of this procedure will end up increasing the risk of intubation failure and complications such as VAP. The other possible reason might be unplanned extubation; the most common cause of reintubation was associated with more prolonged MV duration and ICU stays (64). The high reintubation rate in our study setting, which in turn might be caused by the lack of a well-established comprehensive extubation protocol, could also be the reason.

According to this study, sedation use was revealed as an important factor significantly associated with the mortality of mechanically ventilated patients. The odds of mortality among patients who used sedation decreased by 59% compared to those who were not sedated. This finding is supported by a previous study conducted in Ethiopia (20). This might be because if agitated patients do not get sedated, they might extubate themselves, fight against restraints, and increase the risk of injury. The work of breathing also increases, thereby standing against the main goal of MV and delaying recovery. Wise use of sedation guided by the Richmond Agitation-Sedation Scale assists in the control of sedation, which favors patient care and recovery as well as guides nurses’ decision-making (68). Contrary to this, a study in Egypt (14) showed that patients who used sedatives for 24 h or more had higher odds of mortality than those who did not. This might be due to the intensity of the sedation. Sedation intensity independently, in an ascending relationship, predicted an increased risk of death, delirium, and delayed time to extubation (69). The deeper the patient is sedated, the higher the risk of death and delayed extubation. Many researchers concur that keeping sedation levels equivalent to the Richmond Agitation Sedation Scale, 0, is a clinically desirable goal. They suggest adequately sedating mechanically ventilated patients while balancing against the known negative consequences of excessive sedation (70–73).

### Limitations of the study

Since secondary data review was used, socio-economic, personal, nutritional status, and other socio-demographic characteristics were not explicitly included in this study. The effects of some important predictor parameters of mortality, such as APACHE, SOFA, and qSOFA, were not determined due to the inapplicability of the scores.

## Conclusion

The overall magnitude of mortality in mechanically ventilated patients was high. The main factors associated with increased mortality were advanced age, sepsis, and reintubation history. Hence, special attention to the elderly, patients with sepsis, and reintubated patients could minimize mortality. However, sedation use was found to be associated with decreased mortality odds. In order to calculate and utilize severity scores in the ICU, we recommend having investigation materials such as arterial blood gas analysis in these hospitals. Strengthening the use of sedation scales such as the Richmond agitation sedation scale for MV patients is recommended, and it is better to set a well-established systematic and comprehensive extubation protocol to decrease mortality of mechanically ventilated patients.

## Data availability statement

The raw data supporting the conclusions of this article will be made available by the authors, without undue reservation.

## Ethics statement

The studies involving humans were approved by the School of Nursing Ethical Review Committee at College of Medicine and Health Sciences, University of Gondar. The studies were conducted in accordance with the local legislation and institutional requirements. Written informed consent for participation was not required from the participants or the participants’ legal guardians/next of kin in accordance with the national legislation and institutional requirements.

## Author contributions

ET: Writing – original draft, Software, Resources, Investigation, Formal analysis, Conceptualization. AT: Writing – review & editing, Methodology, Investigation, Conceptualization. NY: Writing – review & editing, Methodology, Investigation, Conceptualization. TN: Writing – review & editing, Software, Investigation, Formal analysis, Data curation. TM: Writing – review & editing, Supervision, Methodology, Investigation.
